# A rare case of follicular lymphoma transformed to a high‐grade B‐cell lymphoma in orbit

**DOI:** 10.1002/ccr3.2153

**Published:** 2019-04-16

**Authors:** Raju K. Vaddepally, Amal Hejab, Vrushali Dabak, Madhu Menon

**Affiliations:** ^1^ Hematology/Oncology Henry Ford Health System Detroit Michigan; ^2^ Internal Medicine Henry Ford Health System Detroit Michigan; ^3^ Hematopathoogy Henry Ford Health System Detroit Michigan

**Keywords:** chemotherapy, follicular lymphoma (FL), monoclonal antibodies, non‐hodgkin lymphoma (NHL), ocular lymphoma, transformation

## Abstract

Transformation of lymphoma is an infrequent phenomenon, and involvement of the eye as such is even uncommon. Histological transformation in patients with follicular lymphoma who were previously treated with immune‐chemotherapy carry a poor outcome. Here, we illustrate such a case with aggressive histological transformation from a low‐grade lymphoma.

## 1 INTRODUCTION

Follicular lymphoma (FL) is an indolent subtype of non‐Hodgkin lymphoma (NHL). Transformation of FL (TL) to an aggressive lymphoma is a well‐recognized phenomenon. FLs most commonly transform to diffuse large cell lymphoma (DLBCL).^1^ Based on literature search, FLs transforming to lymphoblastic leukemia/lymphoma with a double‐hit or triple‐hit (ie, *MYC* translocation with *BCL2* and/or *BCL6* rearrangement) is an extremely rare phenomenon.^2^ Lymphoma involvement of the eye is uncommon,^3^ and to the best of our knowledge, this is the first reported case of a FL transforming to a double‐hit (*MYC* and *BCL2* rearrangement) B‐lymphoblastic lymphoma.

## 2 CASE

A 66‐year‐old man with extensive lymphadenopathy (chest, abdomen, and inguinal lymph nodes) and splenomegaly was diagnosed with FL, predominantly grades 1‐2 with focal areas of grade 3A, Stage IV, with high‐risk FL International Prognostic Index 2 (FLIPI2) score. He had a good response to bendamustine and rituximab. Three months into his treatment, he noticed left eye swelling and visual disturbance. Clinical examination revealed erythematous left orbit with restricted extra‐ocular movements and elevated Intraocular pressure (IOP). MRI was highly concerning for orbital cellulitis with a secondary anterior displacement of left globe. In the emergency room, ophthalmology performed canthotomy and cantholysis to decrease IOP and broad‐spectrum antibiotics were initiated for suspected orbital cellulitis.

**Figure 1:**
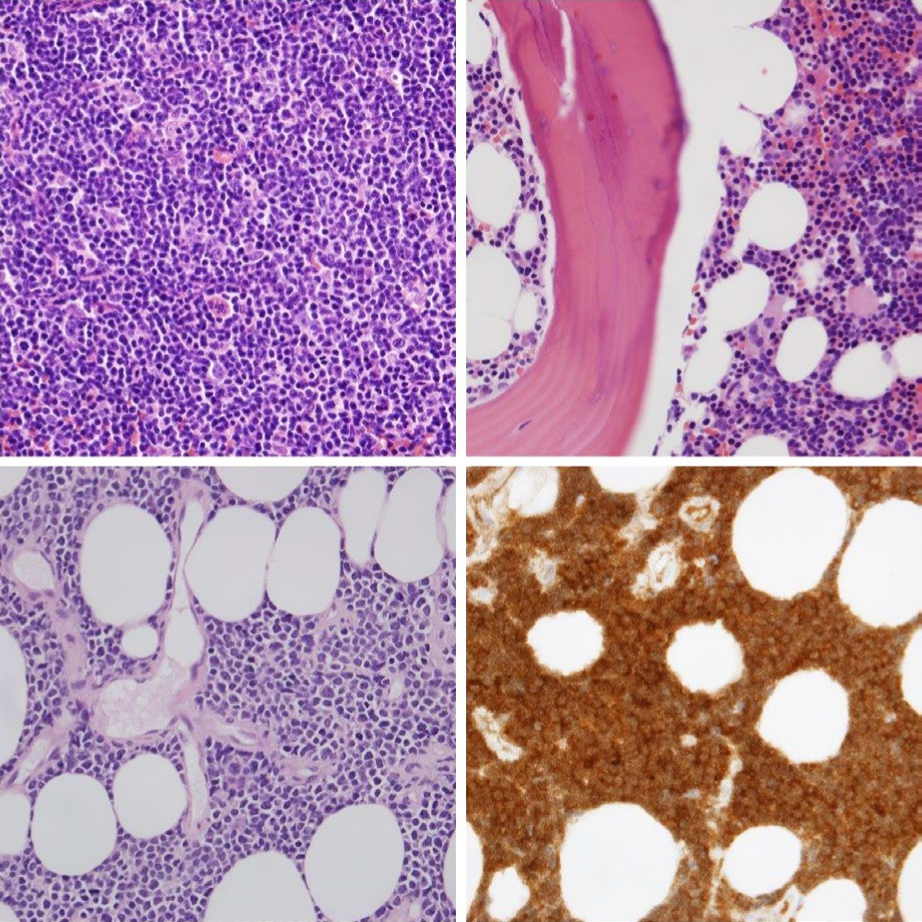
Left upper panel H&E of lymph node biopsy and right upper panel H&E of bone marrow biopsy showing follicular lymphoma. Left lower panel and right lower panel showing H&E and TdT of the ocular biopsy showing lymphoma

On the following day, orbitotomy revealed a firm mass compressing the optic nerve. An excisional biopsy of this mass was obtained. Subsequently, the patient received steroids due to optic nerve compression and antibiotics were discontinued. He later received radiation therapy the following day. Excisional biopsy of the orbital mass revealed a B‐lymphoblastic lymphoma (Table 1; Figure 1), entirely comprising of blastoid B cells, positive for CD19, CD10, TdT, CD38, negative for CD3, CD5, CD11c, and CD20 with Kappa light chain restriction (partial, very dim). Cytogenetics/FISH analysis also demonstrated simultaneous presence of *MYC* and *BCL2* translocation. In light of the patient's history of FL, this B‐lymphoblastic leukemia/lymphoma is presumably a transformed lymphoma. Further diagnostic workup showed the cerebrospinal fluid involvement by B‐lymphoblastic lymphoma. There was no evidence of lymphoma/leukemia in the bone marrow. Subsequently, he received intrathecal cytarabine and methotrexate for leptomeningeal carcinomatosis. He was also started on systemic chemotherapy with HyperCVAD (cyclophosphamide, Vincristine, Adriamycin, and dexamethasone). Unfortunately, the patient could not tolerate intensive chemotherapy after two cycles of therapy and decided to enroll in hospice care.

**Table 1:** Flow cytometry markers

	Original follicular lymphoma	Subsequent B‐lymphoblastic lymphoma
Positive	CD20, CD19, CD10, CD38	CD10, CD19, CD38
Negative	For CD3, CD5, CD11c	CD3, CD20, CD5 and CD11c
Light chain restriction	Kappa restricted	Kappa restricted (partial, very dim)

## 3 DISCUSSION

Transformation of lymphoma (TL) is an infrequent phenomenon and has been reported in approximately 30% of FL.^2^ TL also occurs in marginal zone lymphoma and lymphoplasmacytic lymphoma.^4^ The transformation of FL to a B‐lymphoblastic lymphoma along with a double‐ or triple‐hit is extremely rare and carries dismal prognosis. Histological transformation in patients with FL who previously treated with immunochemotherapy might carry a poor outcome that may deserve intensive salvage therapy including autologous stem cell transplant.^5^

### 3.1 The incidence of TL and its outcomes and relation to rituximab

Several large studies reported the incidence of TL in FL, mainly in the prerituximab era, showing a 5‐ and 10‐year risk of 15% and 30%, respectively, with an estimated incidence rate of 3% per year. The outcome for patients with TL was poor with median survival of 1‐2 years and reported 10‐year survival for patients with nontransformed FL of 75%, as opposed to 36% in patients with evidence of transformation.^6^, ^7^

After the wide adoption of rituximab in the management of NHL, the incidence and outcomes of TL were restudied. The incidence of TL was ranging between 2% per year^8^ and 6% every 2 years.^9^ Initial treatment with rituximab was associated with significantly reduced transformation risk vs patients who were only initially observed (3.2% vs 14.4%, respectively).^8^, ^9^ Survival of TL in the postrituximab era is evidently better than what was reported in prerituximab era. Wagner et al^9^ reported that the 5‐year survival for patients in the postrituximab era is significantly improved, TL had a survival rate of 75%, and nontransformed lymphomas had survival of 85%.

### 3.2 Clinical prognostics of TL

Studies have been done to determine prognostic factors in patients with TL in prerituximab era. Some of the indicators for worse survival include high lactate dehydrogenase (LDH), advanced stage disease, and not having achieved complete remission.^6^ However, these factors did not have similar prognostic value in postrituximab era^8^, ^9^ thus needing further exploration.

### 3.3 Diagnosis of TL

Transformation of lymphoma is generally diagnosed by histology. Transformed FL may or may not retain markers similar to its original follicular origin. Although changes in the antigenic marker are common, the light chain restriction is usually preserved. Therefore, a change of the light chain restriction from kappa to lambda or vice versa should raise the concern for a second malignancy rather than TL.^1^

Less commonly, clinical diagnosis of TL can be made, if the histological diagnosis is not feasible, based on reliable predictors such as rising LDH, rapid nodal growth, the involvement of new extranodal sites, sudden decline in performance status, new “B” symptoms and hypercalcemia.^6^, ^8^, ^10^ Radiological studies, such as positron emission tomography (PET‐CT) scans, could be used in this regard. For PET‐CT scans, higher standardized uptake values (SUVs) more than 13 is predictive of an aggressive lymphoma (sensitivity of 90%). In addition, the positive predictive value for detecting TL is 100%, if SUV max is more than 17.^11^

### 3.4 TL of follicular lymphoma to B‐lymphoblastic leukemia/lymphoma

Acquiring MYC rearrangement is a detrimental step contributing to the development of transformed lymphoma.^2^, ^12^, ^13^ Based on literature search, lymphoblastoid transformation of FL is a rare phenomenon, and Geyer et al^2^ reported a series of seven cases with an additional review of additional 20 cases reported in the literature, noting the various names it was given: Acute lymphoblastic leukemia, precursor B‐cell blast crisis, and atypical Burkitt lymphoma. Most FL cases carry t(14;18) involving *BCL2* gene. Most reported cases (upward of 90%) of lymphoblastoid transformation of FL are double‐hit (ie, carry both *MYC* and *BCL2* gene rearrangement). While the prognosis of lymphoblastoid transformation with double‐hit is slightly worse than that of de novo double‐hit lymphoma, its unclear whether the lymphoblastoid morphology or the double‐hit nature of this transformation confers a worse prognosis than what would be expected from a typical tranformation of FL to DLBCL.^2^ Geyer et al suggested the unifying term of “lymphoblastic transformation of FL.” However, the current WHO recommended terminology for either de novo double‐hit blastoid TdT+ lymphoma or FL transforming to blastoid TdT+ double‐hit lymphoma is “lymphoblastic”.^14^

### 3.5 Ocular lymphoma

Ocular Lymphoma is the most frequent tumor of all malignant ocular tumors, constituting up to 55% of all orbital tumors.^15^ Ocular lymphomas are broadly separated into two main categories: intraocular lymphoma, which is mainly a subset of primary central nervous system lymphoma (IOL), and ocular adnexal Lymphoma (OAL), which are mainly NHL. This classification is important because of the great differences in natural history, management options, and outcomes. OAL can present in many different ways such, as an orbital mass, a lesion of the lacrimal gland or less frequently it can involve the conjunctiva or the eyelids. Very rarely, OAL may present as intraocular lymphoma.^16^ Of all the OAL, marginal zone B‐cell lymphoma (MZL) and mucosa‐associated lymphoid tissue (MALT) lymphoma comprise approximately 50%. The other two more common reported subtypes of OAL include lymphoplasmacytic and follicular lymphomas.^17^ The outcomes for OAL is generally favorable with 5‐yearsoverall survival rate ranging from 50% to 94%. These data are mainly driven by the reported outcomes of OAL‐MALT lymphoma. OAL has an overall outcome that is similar to that of its cousin, nodal NHL, namely better outcomes in lower grades disease. This similarity of ocular adnexal and its more common cousin, nodal lymphoma, implies no real prognostic implications of initial Ocular lymphoma involvement.^16^

### 3.6 Management of TL

The management of TL is mainly derived from the evidence of treating FL transformed to DLBCL. The treatment, in general, includes rituximab‐containing high‐dose chemotherapy, with and without autologous hematopoietic stem cell transplant. It has been shown that intensification of regimens, including transplant, can improve survival.^18^ Currently, new methods are being explored to treat TL, one of which is radioimmunotherapy using radioisotopes labeled monoclonal antibodies, which seems to be promising.^19^

The management of ocular adnexal lymphoma on the other hand, given its anatomical involvement, generally involves radiotherapy. While radiotherapy is the most extensively employed approach, the response rates are usually low. Other management approaches include chemotherapy, immunotherapy, and surgical excision followed by observation.^16^ In our case, given the aggressive nature of this transformed lymphoma, we adopted a combination of radiation therapy to the orbit and high‐dose chemotherapy (HyperCVAD).

## 4 CONCLUSION

Transformation of indolent lymphoma to more aggressive lymphoma is an infrequent event and carries a poor prognosis. TL isolated to the orbit is extremely uncommon. To the best of our knowledge, this is the first case report of such transformation of FL to B‐lymphoblastic lymphoma with a simultaneous *MYC* and *BCL2* translocation (double‐hit) in the eye. A presentation of what seems like orbital cellulitis in patients with indolent lymphoma may, in fact, be a presentation of ocular lymphomatous involvement.

## CONFLICT OF INTEREST

None declared.

## AUTHOR CONTRIBUTIONS

RKV: involved in conception and design of work, drafting, and final approval of the version. AH: drafted the manuscript and collected data. VD: involved in revision of the article. MM: involved in critical revision of article.
